# Nogo-Receptors NgR1 and NgR2 Do Not Mediate Regulation of CD4 T Helper Responses and CNS Repair in Experimental Autoimmune Encephalomyelitis

**DOI:** 10.1371/journal.pone.0026341

**Published:** 2011-11-11

**Authors:** Karin Steinbach, Claire L. McDonald, Markus Reindl, Rüdiger Schweigreiter, Christine Bandtlow, Roland Martin

**Affiliations:** 1 Institute for Neuroimmunology and Clinical MS-Research, Hamburg, Germany; 2 Clinical Department of Neurology, Innsbruck Medical University, Innsbruck, Austria; 3 Department of Neurobiochemistry, Innsbruck Medical University, Innsbruck, Austria; Julius-Maximilians-Universität Würzburg, Germany

## Abstract

Myelin-associated inhibition of axonal regrowth after injury is considered one important factor that contributes to regeneration failure in the adult central nervous system (CNS). Blocking strategies targeting this pathway have been successfully applied in several nerve injury models, including experimental autoimmune encephalomyelitis (EAE), suggesting myelin-associated inhibitors (MAIs) and functionally related molecules as targets to enhance regeneration in multiple sclerosis. NgR1 and NgR2 were identified as interaction partners for the myelin proteins Nogo-A, MAG and OMgp and are probably mediating their growth-inhibitory effects on axons, although the *in vivo* relevance of this pathway is currently under debate. Recently, alternative functions of MAIs and NgRs in the regulation of immune cell migration and T cell differentiation have been described. Whether and to what extent NgR1 and NgR2 are contributing to Nogo and MAG-related inhibition of neuroregeneration or immunomodulation during EAE is currently unknown. Here we show that genetic deletion of both receptors does not promote functional recovery during EAE and that NgR1 and NgR2-mediated signals play a minor role in the development of CNS inflammation. Induction of EAE in Ngr1/2-double mutant mice resulted in indifferent disease course and tissue damage when compared to WT controls. Further, the development of encephalitogenic CD4^+^ Th1 and Th17 responses was unchanged. However, we observed a slightly increased leukocyte infiltration into the CNS in the absence of NgR1 and NgR2, indicating that NgRs might be involved in the regulation of immune cell migration in the CNS. Our study demonstrates the urgent need for a more detailed knowledge on the multifunctional roles of ligands and receptors involved in CNS regeneration failure.

## Introduction

The non-regenerative nature of the adult mammalian central nervous system (CNS) poses a major challenge to successful repair of nerve damage occurring by either traumatic injury or during inflammatory CNS diseases such as Multiple Sclerosis (MS). Most likely driven by a deregulated myelin-specific autoreactive CD4^+^ T cell response, this disease leads to chronic inflammation, demyelination, and neuronal and axonal degeneration [1], [2]. The latter two outcomes are considered to be the major determinants of clinical disability in patients [3], [4], [5]. Axonal regrowth and plasticity in the adult is limited by several, probably redundant regulatory pathways including inhibitory proteins of the CNS myelin [6], formation of a glial scar upon injury [7] as well as lack of intrinsic growth capacity in CNS neurons [8].

Nogo receptors were identified as interaction partners for three myelin proteins associated with the inhibition of axonal regeneration in the adult mammalian CNS (MAIs) – Nogo, myelin-associated glycoprotein (MAG) and oligodendrocyte-myelin glycoprotein (OMgp) [9], [10], [11]. While NgR1 serves as common receptor for the Nogo-66 inhibitory domain common to all three isoforms of Nogo, Nogo-A, -B and -C, as well as MAG and OMgp; NgR2 was shown to be binding partner for MAG [9], [10], [11], [12]. Together with paired-immunoglobulin-like receptor B (PirB) [13] and probably other mechanisms [14], [15], signalling via NgR1, NgR2 and coreceptors induces growth cone collapse and inhibition of axonal regrowth as well as compensatory sprouting of remaining axons, thereby impairing functional repair after injury. However, although many components of this regulatory system have been identified by extensive and detailed studies, their relative contribution to CNS regeneration failure *in vivo* is still poorly understood.

Furthermore, alternative functions for NgR1 and NgR2 in the regulation of nervous tissue damage recently emerged when a potential immunoregulatory role for NgRs in inflammatory responses was described. Although both receptors are only weakly expressed on naive immune cells, upregulation of NgR1 and NgR2 over time can be detected on several immune cell types after *in vitro* stimulation [16], as well as *in vivo* in models of nerve injury [17] and in MS lesions [18]. Upregulation of NgR1 and NgR2 was shown to induce repulsion from myelin substrates *in vitro* leading to efflux from the injured peripheral nervous system (PNS). Although a similar function has been suggested for the CNS [19], it is so far unknown, whether NgR1 and NgR2 regulate leukocyte migration in the CNS *in vivo*.

Evidence for a disease-modifying role of MAIs in MS is provided by studies in the established animal model, experimental autoimmune encephalomyelitis (EAE). Blockade of Nogo leads to an ameliorated disease course with enhanced functional recovery associated with less permanent axonal damage [20], [21], [22]. Interestingly, some approaches also resulted in an altered myelin-specific T cell response in the treated animals, supporting an immunoregulatory role for Nogo in addition to its inhibitory function on CNS regeneration. So far it has not been studied whether the immunomodulatory effects of Nogo are indeed provoked by the same receptors, e.g. NgR1, that mediate its inhibitory functions in the CNS.

In order to understand better the potential multifunctional roles for NgR1- and NgR2-mediated signals in the development of inflammatory responses and the occurrence of inflammation-induced neuronal and axonal damage and regeneration in the CNS, we studied MOG_35–55_-induced chronic EAE in *Ngr1* ^−/−^ [23], *Ngr2* ^−/−^ and *Ngr1/2* ^−/−^ double mutant mice [24]. Here we provide evidence that genetic deletion of NgR1 and NgR2 has only minor effects on the development of inflammatory responses in the CNS and does not improve inflammation-induced neuronal and axonal damage in this model. However, leukocyte infiltration was slightly enhanced in the CNS of *Ngr1/2* ^−/−^ double mutant mice. Further, the lack of immunomodulatory effects in double mutant mice indicates that the Nogo-66 receptor NgR1 is not involved in potential immunoregulatory functions of Nogo proteins.

## Results

### NgR-deficiency does not alter the clinical disease course of EAE

**Figure 1:**
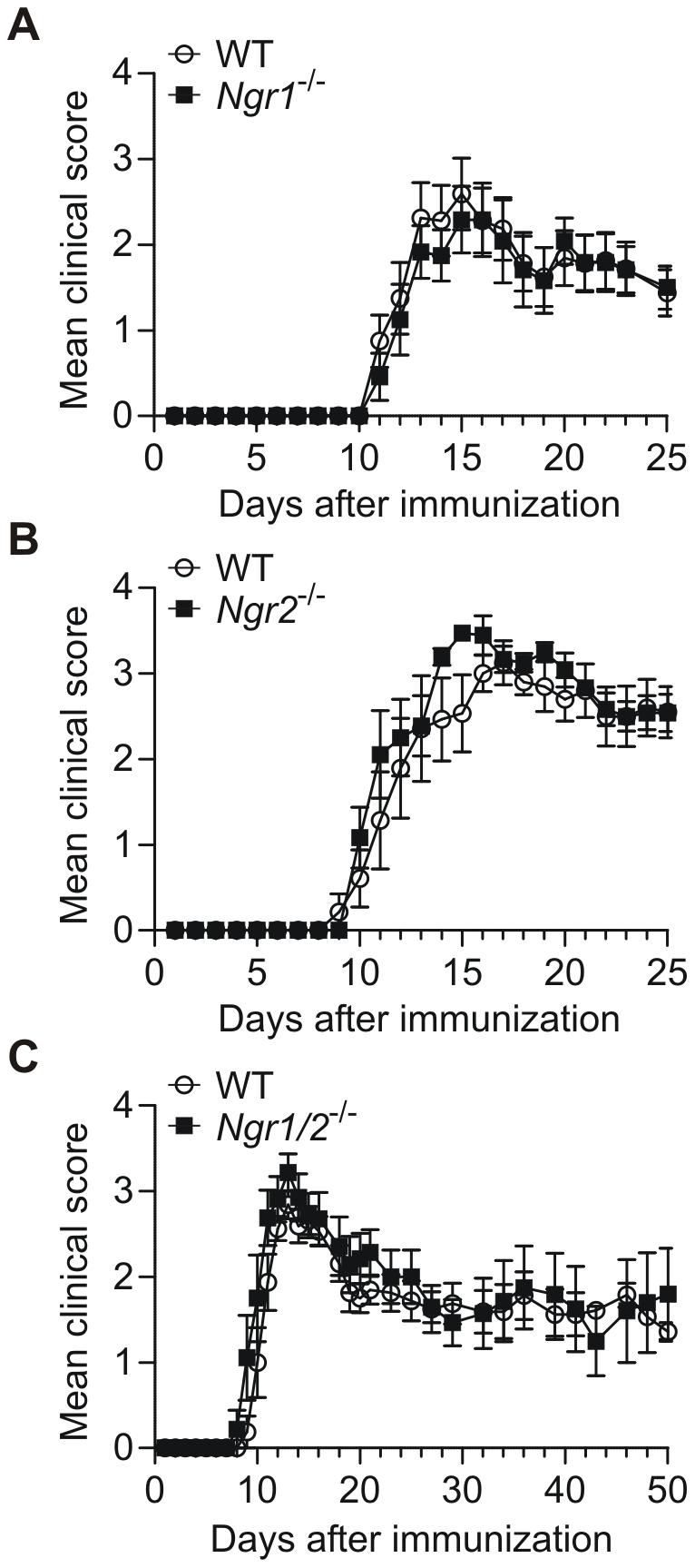
Clinical course of EAE in Nogo receptor-deficient animals. EAE was induced in *Ngr1* ^−/−^ (A), *Ngr2* ^−/−^ (B) and *Ngr1/2* ^−/−^ animals (C), and disease course was monitored in comparison to WT controls for at least 30 days as described in materials and methods. One representative experiment out of at least two is shown. Clinical scores represent mean ± s.e.m. of diseased animals (n≥6; see also table 1).

In order to investigate a potential role for NgR1 and NgR2 in the development of inflammatory responses in the CNS and/ or recovery from the resulting neuronal and axonal damage, we induced EAE in different NgR-deficient animals and compared their clinical course to corresponding WT controls (Fig. 1, table 1). Neither *Ngr1* ^−/−^ (Fig. 1A), *Ngr2* ^−/−^ (Fig. 1B) nor *Ngr1/2* ^−/−^ mice (Fig. 1C) showed a significantly altered disease course compared to corresponding WT mice, although we did observe a trend towards slightly enhanced mean clinical scores during acute EAE at days 13–15 for *Ngr2* ^−/−^ and *Ngr1/2* ^−/−^ mice in all experiments performed. Furthermore, there was no significant difference between NgR-deficient mouse lines and corresponding WT controls when analysed with respect to disease incidence, day of disease onset or maximal disease severity (Table 1). Of note, *Ngr1/2* ^−/−^ mice showed an increased mortality rate over a 50-day observation period, which could be an additional indicator of a potential aggravation of acute EAE in these mice. However, since deletion of NgR1 and NgR2 did not lead to an increased mortality during the acute stages of EAE, we conclude that NgR1 and NgR2 apparently do not play a major role in the development of CNS inflammation and long-term disease progression during EAE.

**Table 1:** Clinical course of EAE in Nogo receptor-deficient animals.

	WT	Ngr1^−/−^	WT	Ngr2^−/−^	WT	Ngr1/2^−/−^
Incidence	7/8	6/8	7/8	9/9	8/11	9/10
Mean day of onset^A^	11.4±0.3	11.7±0.3	11.7±0.9	11.0±0.5	10.2±0.3	9.8±0.4
Max. clinical score^A^	3.1±0.2	2.5±0.3	3.6±0.1	3.8±0.1	3.0±0.1	3.3±0.2
Mortality rate	1/8	0/8	2/8	3/9	1/11	4/10

Disease parameters of representative experiments presented in figure 1 are shown. Results represent mean ± s.e.m.

A : Diseased animals only.

### NgR-deficiency does not improve axonal and neuronal loss during EAE

**Figure 2:**
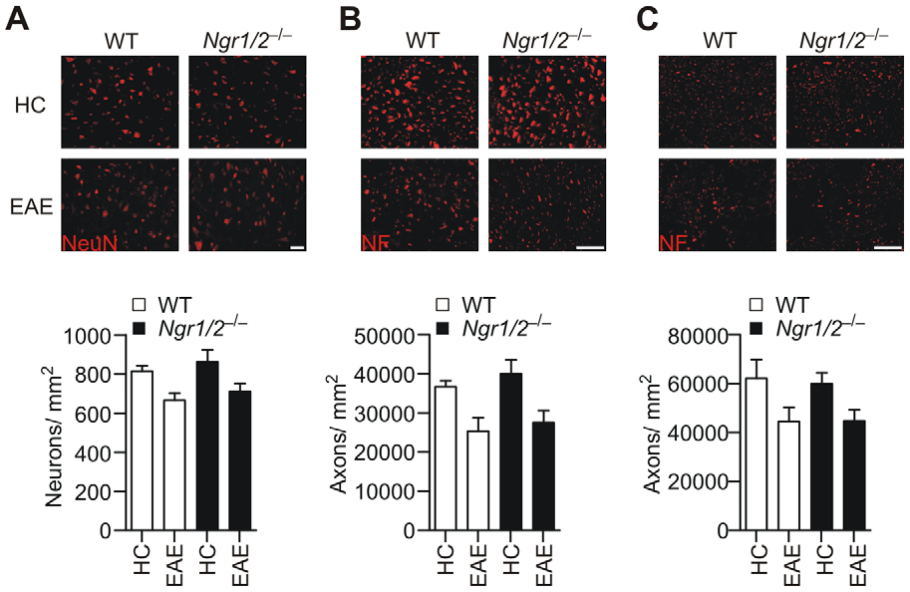
Neuronal and axonal damage in *Ngr1/2* ^−/−^ mice. Neuronal and axonal loss was quantified in WT and *Ngr1/2* ^−/−^ animals 30 days after EAE induction and compared to unimmunised healthy controls (HC). Cell bodies of ventral horn motor neurons were stained with anti-NeuN antibody and neuronal nuclei were counted in cervical spinal cord sections (A). Scale bars represent 50 µm. Loss of neuronal nuclei in chronic EAE was statistically significant (p<0.01), while differences between genotypes was not (p = 0.35; two-way ANOVA). Axons in the dorsal column (B) and corticospinal tract (C) were stained with anti-neurofilament (NF) antibodies SMI-31 and SMI-32 and quantified as described in material and methods. Axonal loss in chronic EAE was statistically significant (p<0.01 for DC, p<0.05 for CST), while differences between genotypes was not (p = 0.42 for DC axons, p = 0.86 for CST axons; two-way ANOVA). Scale bars represent 20 µm. Representative staining images are shown. Results are mean ± s.e.m.. (n≥3 for HC and n = 7 for diseased animals).

Based on the known functions of NgR1 and NgR2 as mediators of myelin-associated inhibition of axonal regrowth, we hypothesized that NgR-deficiency might result in enhanced repair of CNS damage. This, however, does not necessarily have to translate into a significant alteration of clinical symptoms during chronic EAE. In order to determine if *Ngr1/2* ^−/−^ mice show enhanced recovery at the cellular level, neuronal and axonal loss in the spinal cord of chronic EAE mice was quantified (Fig. 2). Thirty days after EAE induction, axonal and neuronal loss was evident in cervical spinal cord sections in comparison to unimmunised control animals (p<0.01 for ventral horn neuronal nuclei and axons of the dorsal column; p<0.05 for axons of the corticospinal tract). Corresponding to the similar clinical courses during chronic EAE, the loss of ventral horn motor neurons (Fig. 2A) and axons in the dorsal column (Fig. 2B), as well as in the corticospinal tract (Fig. 2C) was unchanged between *Ngr1/2* ^−/−^ and WT mice. Thus, deletion of NgR1 and NgR2 does not result in enhanced repair or recovery from EAE at the cellular and functional level.

### NgR-deficiency does not influence the encephalitogenic immune response during EAE

**Figure 3:**
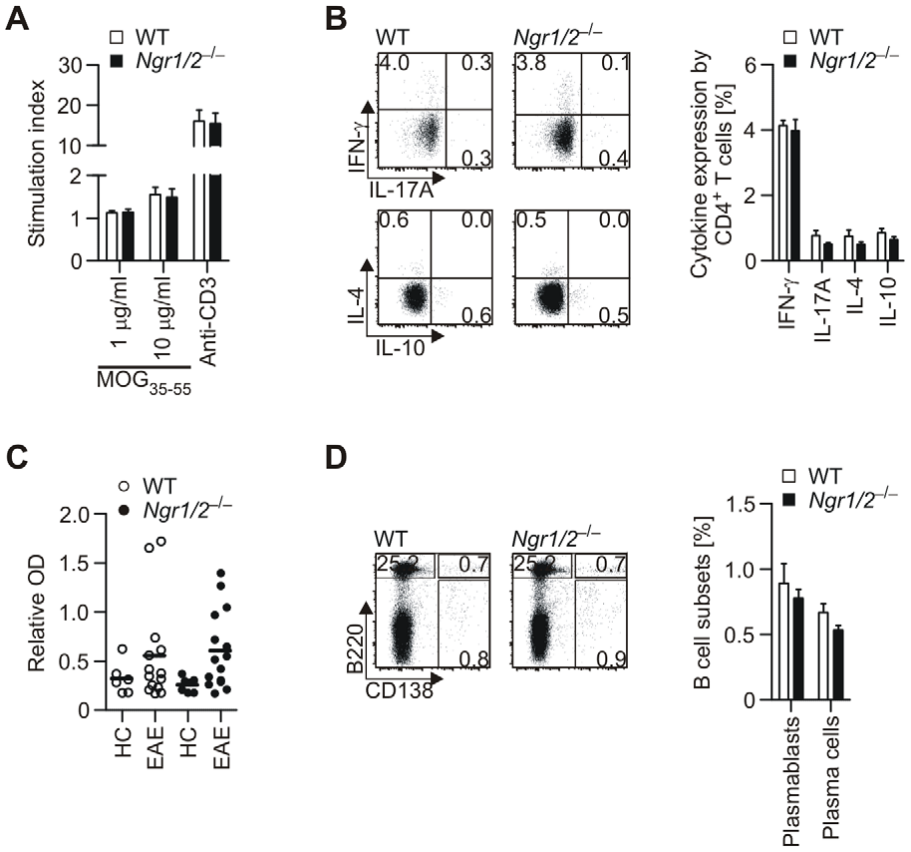
Peripheral immune response in *Ngr1/2* ^−/−^ mice. Analysis of peripheral T cell response (A–B): Single cell suspensions from draining lymph nodes of immunized *Ngr1/2* ^−/−^ or WT mice were restimulated *in vitro* with either MOG 35–55 peptide or anti-CD3 and resulting T cell proliferation was assessed by ^3^H-thymidine incorporation (A). Cytokine production by CD4^+^ T cells was assessed by intracellular cytokine staining after *ex vivo* restimulation with PMA/ Ionomycin. Representative staining images are shown (B). Analysis of peripheral B cell activation (C–D): Anti-MOG antibody titres in serum were quantified at peak of disease (n≥14) in comparison to healthy controls (HC, n≥6) as described in material and methods (C). Activated B cells and plasma cells were quantified by flow cytometry in splenocytes. Representative staining images are shown (D). Results represent mean ± s.e.m. (n≥5).

In line with previous studies [16], [17] we were able to detect NgR1 and NgR2 mRNA expression in T cells, B cells and myeloid cells from mouse and human origin [25]. Since the myelin-specific immune response in MOG-immunized Nogo deletion mutants was shown to be altered in favour of an anti-inflammatory response associated with Th2-cytokines [20], we started to investigate the peripheral immune response to immunisation with MOG peptide in *Ngr1/2* ^−/−^ mice (Fig. 3). We observed a similar recall response of *in vitro* restimulated T cells to MOG 35–55 peptide (Fig. 3A), which was not associated with a change in production of pro- or anti-inflammatory cytokines (data not shown). Accordingly, we detected similar frequencies of IFN-γ-producing Th1 cells, IL-17A-producing Th17 cells, IL-4-producing Th2 cells or IL-10-producing CD4^+^ T cells in the spleens of *Ngr1/2* ^−/−^ and WT mice after immunization (Fig. 3B), indicating that the peripheral T cell response is not substantially altered. NgR1 has been shown to interact with B cell-activating factor (BAFF) [26], which promotes B cell development as well as B cell activation and differentiation into antibody-producing cells [27], [28], [29]. Since the absence of NgR1 and NgR2 could result in an enhanced availability of BAFF to B cells, we additionally investigated the peripheral B cell response in *Ngr1/2* ^−/−^ mice (Fig. 3C–D). However, anti-MOG antibody titers (Fig. 3C) as well as the frequencies of antibody-producing cells (plasma cells, plasma blasts and B cells; Fig. 3D) were similar in immunized *Ngr1/2* ^−/−^ and WT mice.

These results indicate that the previously observed shift of the peripheral immune response in Nogo deletion mutants [20], [21] is not due to a release from NgR1 and NgR2-mediated signals, and that both receptors are dispensable for the priming of a peripheral T- and B-cell response upon immunization with CFA.

### NgR-deficiency does not significantly change CNS inflammation during EAE

**Figure 4:**
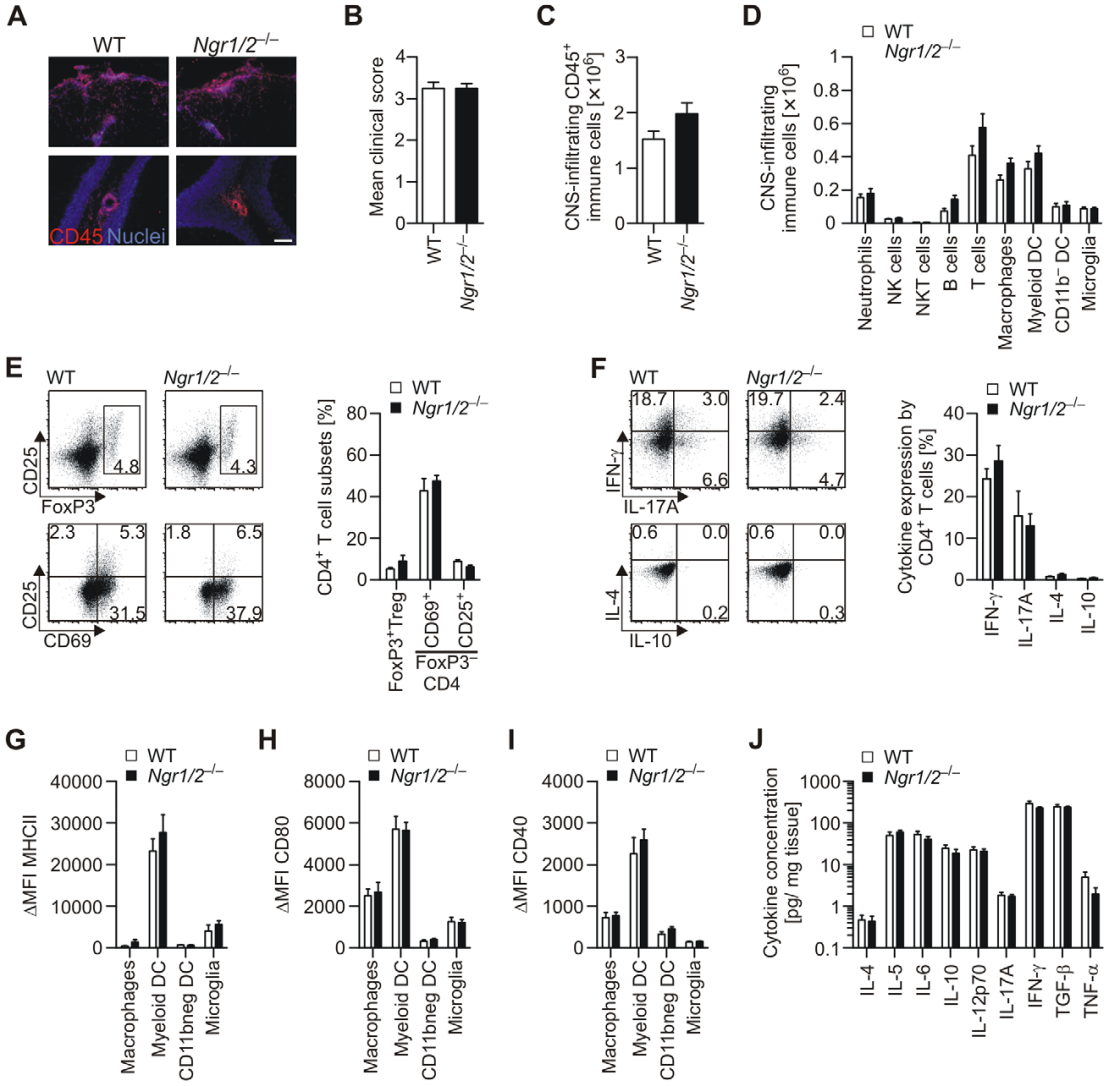
CNS inflammation in *Ngr1/2* ^−/−^ mice at peak of disease. Representative immunohistochemical staining of CD45-positive leukocytes in *Ngr1/2* ^−/−^ mice and WT controls in cervical spinal cord (upper panel) and cerebellum (lower panel) (A). Flow cytometric quantification of CNS-infiltrating cells from *Ngr1/2* ^−/−^ mice and WT controls (B–D): Mean clinical scores of analysed mice (B). Numbers of CD45^+^ CNS-infiltrating cells (p = 0.07, unpaired student's t-test) (C) and different immune cell types within CD45^+^ cells (p<0.01 for T cells, two-way ANOVA combined with Bonferroni post-analysis) (D). Results are pooled from three independent experiments (n≥13). Flow cytometric analysis of CNS-infiltrating CD4^+^ T cells (E–F). Frequencies of FoxP3^+^ Tregs (upper panel) and expression of activation markers CD69 and CD25 on FoxP3^−^ effector T cells (lower panel) were quantified. Representative staining images are shown (n = 3) (E). Cytokine production by CD4^+^ T cells was assessed by intracellular cytokine staining after *ex vivo* restimulation with PMA/ Ionomycin. Representative staining images are shown (n = 3) (F). Flow cytometric analysis of APC (G–I). Median fluorescence intensity of the maturation markers MHCII (G), CD80 (H) and CD40 (I) was analysed on indicated cell populations in comparison to control stainings (n = 3). Cytokine concentration in the CNS of *Ngr1/2* ^−/−^ mice and WT controls during acute EAE was analyzed in whole brain homogenates as described in material and methods (n≥8) (J). Results represent mean ± s.e.m. for all shown results.

NgR1 and NgR2 have been implicated in the regulation of immune cell migration into nervous tissue, particularly in the PNS [17], and a similar function has been suggested for inflammatory responses in the CNS [19]. Therefore we investigated whether NgR1 and NgR2 can influence the recruitment of inflammatory cells to the CNS during acute EAE (Fig. 4). Since we did not observe striking alterations in the size and distribution of acute inflammatory lesions in the CNS of *Ngr1/2* ^−/−^ mice in comparison to WT mice (Fig. 4A), we performed a detailed flow cytometric analysis of the overall immune cell infiltration into the CNS during acute disease (Fig. 4B–D). Although the analyzed groups of *Ngr1/2* ^−/−^ and WT mice have similar mean clinical scores (Fig. 4B), the number of CNS-infiltrating CD45^+^ immune cells was slightly increased in *Ngr1/2* ^−/−^ mice in all three experiments performed, although this did not reach statistical significance (p = 0.07 in pooled data). Further, the increase in CNS-infiltration by immune cells could not be attributed to a single cell type, but was rather mediated by an overall increased recruitment of different immune cells, which reached statistical significance for T cells (p<0.01 in pooled data) (Fig. 4D). This indicates a general influence of NgR1 and NgR2 on immune cell migration into the CNS, although the observed effects appear marginal and probably do not influence disease severity in *Ngr1/2* ^−/−^ mice (compare Fig. 1C). Next we investigated whether this observed slight increase in CNS-infiltration might alter the phenotype of the inflammatory response in the CNS (Fig. 4E–J). However, we did not observe any differences in terms of CD4^+^ T cell activation (Fig. 4E), cytokine production (Fig. 4F), or in the expression of maturation markers like MHCII (Fig. 4G), CD80 (Fig. 4H) and CD40 (Fig. 4I) on APCs like macrophages, dendritic cell subsets and microglia. Correspondingly, we detected similar levels of pro- and anti-inflammatory cytokines in the whole CNS extracts (Fig. 4J), indicating that the overall inflammatory milieu in the inflamed CNS is not changed in *Ngr1/2* ^−/−^ mice, even if leukocyte migration into the CNS might be slightly facilitated.

## Discussion

Strategies aiming at the improvement of axonal regeneration and repair are currently in clinical development and are clearly desirable for patients suffering from traumatic injury as well as from diseases like MS, which is associated with axonal transections, neuronal loss and consequently chronic disability accumulation. However, the interactions and pathways between inhibitors of axonal regrowth like MAIs and their receptor molecules are still poorly understood and probably far more complicated than previously appreciated. Here we provide evidence that two proteins involved in the negative regulation of axonal regrowth and plasticity by MAIs, NgR1 and NgR2, are dispensable with respect to maintaining myelin-associated inhibition of axonal regrowth in the development and progression of autoimmune inflammatory neurodegeneration in EAE, an established animal model of the human disease MS.

Using several blocking strategies against Nogo-A, a potent inhibitor of axonal regeneration, a number of studies reported beneficial effects, which could in part be attributed to enhanced repair mechanisms [20], [21], [22]. We now demonstrate that combined genetic deletion of the Nogo-66 receptor NgR1 and one receptor for MAG, NgR2, did not result in enhanced repair and functional recovery from inflammation-induced axonal damage in a chronic model of EAE. In spite of the established role of NgR1 and NgR2 in mediating growth-inhibition of Nogo-66 and MAG *in vitro*, genetic deletion of both receptors did not result in enhanced axonal density in the corticospinal tract and dorsal column in EAE. Since common inhibition of axonal regrowth by Nogo-66, MAG and Omgp is at least mediated by one additional receptor, PirB [13], and additional inhibitory cues probably exist, genetic deletion of NgR1 and NgR2 appears insufficient to relieve axons from growth-inhibition by myelin and both receptors are probably functionally compensated by PirB and/ or other mechanisms. These data argue that the inhibition of axonal outgrowth in the adult CNS is mediated by complex interactions of multiple receptor-ligands and well secured to the point that even deletion of two important receptors, NgR1 and NgR2, does not result in enhanced axonal sprouting.

As mentioned above, there have been numerous studies focusing on promoting axonal outgrowth in EAE by targeting Nogo via immunisation, antibody treatment or genetic deletion. These studies observed an influence on immune function, i.e. a shift of the myelin-specific T cell response towards a Th2-like (anti-inflammatory) cytokine profile, which is probably partly responsible for the beneficial effects observed in these studies [20], [21]. In contrast, we did not observe alterations in T cell proliferation and cytokine production in *Ngr1/2* ^−/−^ mice, indicating that Nogo is not exerting its potential immunomodulatory functions via the Nogo-66 receptor NgR1. Accordingly, it has been demonstrated that human T cell proliferation and cytokine production is insensitive to treatment with a Nogo-66-derived antagonistic peptide [16], indicating that other receptors than NgR1 mediate immunomodulatory functions by Nogo proteins. In fact, the Th2-like cytokine shift observed in the above-mentioned studies might not even be due to the blockade of Nogo-A, since a recent study specifically targeting Nogo-A during EAE did not find differences in the proliferative capacity, cytokine production or the ability to transfer disease in myelin-specific T cells from treated mice although functional repair and recovery was observed [22]. Instead, the Nogo-B isoform has been implicated in the regulation of immune cell migration [30], [31] and in the regulation of Th2-driven inflammatory reponses in the lung [32]. Further, in contrast to Nogo-A and Nogo-C, Nogo-B is widely expressed in immune cells [33]. It remains to be seen whether the Nogo-B receptor NgBR [34] or the recently identified additional common receptor of Nogo-66, MAG and OMG, PirB, is the responsible interaction partner for these immunoregulatory functions of Nogo-B.

During our investigation of immune cell infiltration into the CNS during acute disease, we observed an increase in the number of infiltrating leukocytes during the acute phase of EAE. Since this increase was not attributable to a single cell type but affected several investigated cells, we conclude that the absence of NgR1 and NgR2 facilitates leukocyte recruitment to the CNS in *Ngr1/2* ^−/−^ mice. Leukocytes expressing NgRs are probably inhibited or even repulsed from migrating into nervous system tissue by myelin, since blockade or silencing of NgRs increases the adhesion of leukocytes to myelin substrates *in vitro* [16], [17] and the efflux of macrophages from injured peripheral nerve tissue is associated with the upregulation of NgR1 and NgR2 *in vivo* [17]. Here we provide evidence that a similar mechanism might regulate immune cell recruitment and/ or spreading in the CNS as has been suggested [19]. NgR1 and NgR2 therefore probably regulate immune cell recruitment to nervous tissue in a generalized manner.

The fact that the increase in leukocyte infiltration into the CNS is not as pronounced as described for injured PNS and has only minor consequences at least in the EAE model could be explained in several ways. First, repulsion of immune cells requires the upregulation of NgR1 and NgR2. The exact conditions of NgR expression on immune cells infiltrating into the CNS remain to be studied. In fact we were not able to detect upregulation of NgR1 or NgR2 in the inflamed CNS tissue during EAE (data not shown), and unfortunately no NgR1 or NgR2-specific antibody was available for our study to perform flow cytometric or immunohistochemical analysis of CNS-infiltrating cells. Additionally, upregulation of NgR expression on immune cells or their repulsive function might be inhibited or overruled by other signals during ongoing inflammatory responses in the CNS. Last but not least, repulsion of NgR-expressing immune cells from myelinated (healthy) CNS areas might not be solely dependent on interactions between MAIs and NgRs. Astrocytes, which are not present in the PNS, have a clear role in restricting the inflammatory lesion and in the prevention of immune cell spreading in the CNS [35]. These and other factors could functionally compensate for the loss of NgR1 and NgR2 and result in differences in the relative importance of NgR-mediated regulation of immune cell migration between the CNS and the PNS. Our data support the hypothesis that NgR1 and NgR2 regulate immune cell migration into nervous tissue. The observed enhanced inflammatory response in *Ngr1/2* ^−/−^ mice demonstrates the urgent need for further studies on the multifunctional roles of ligands and receptors involved in the non-regenerative nature of the adult CNS. Only with a detailed knowledge of all participants will we be able to identify appropriate single or multiple targets in that system, which allow a specific and safe improvement of neuronal and axonal repair and regeneration.

## Materials and Methods

### Ethics statement

All animal experiments were performed in accordance with the guidelines of the local authorities (Behörde für Soziales, Familie, Gesundheit und Verbraucherschutz Hamburg; G07/025 and G08/007).

### Mice

Wildtype C57BL6/J were obtained from the Jackson Laboratory and bred in the animal facility of the University Medical Center Eppendorf. *Rtn4r* ^−/−^ mice (B6.129S7/SvEvBrd-Rtn4r^tm1Matl^) [23] and *Rtn4rl2* ^−/−^ mice (B6-TgH(NgRH1)^143Npa^) [24] have been previously described. These mice are further referred to as *Ngr1* ^−/−^ mice for *Rtn4r* ^−/−^ mice, *Ngr2* ^−/−^ mice for *Rtn4rl2* ^−/−^ mice and *Ngr1/2* ^−/−^ mice (for *Rtn4r/Rtn4rl2* double mutant mice). For all experiments performed, offspring of homozygote matings established from littermate animals were used.

### EAE

Six to 10 week old mice were injected subcutaneously on two spots at the flanks with 100 µl of 200 µg MOG 35–55 emulsified in CFA supplemented with 2 mg/ ml *Mycobacterium tuberculosis H37Ra*. Immunised animals were administered 300 ng of pertussis toxin intravenously the same day and intraperitoneally two days later. EAE developed after approximately 10 days and was scored daily based on a 5-point EAE scale (0: no disease symptoms; 1: limp tail; 2: hind limb paresis; 3: partial hind limb paralysis; 3.5: complete hind limb paralysis; 4: hind limb paralysis and fore limb paresis; 5: moribund or dead). Food and water access for severely disabled animals was assured. Mice with complete hind limb paralysis continuing over 3 days or which suffered from tetraparalysis were euthanized.

### Immunohistochemistry

Mice were anesthesized and transcardially perfused with 4% PFA. Prepared tissue was post-fixed in 4% PFA for 30 min at 4°C and then transferred into 30% sucrose for 24 h. Cervical, thoracic and lumbar spinal cord as well as cerebellum and forebrain were separated, embedded in tissue freezing medium and frozen in isopentane cooled on dry ice. Frozen tissue blocks were stored at −80°C. Cryosections of 14 or 20 µm were sliced at −17°C, mounted onto slides and stored again at −80°C until further use. Cryosections were incubated in blocking solution for 1 h at RT, washed once in PBS and incubated over night at 4°C with anti-CD45 (30-F11), anti-NeuN (A60) or anti-Neurofilaments antibodies (SMI-31 and SMI-32) diluted in PBS. Sections were washed three times in PBS for 5 min and incubated for 1 h at RT with fluorescently labeled secondary antibodies (all from Jackson) diluted in PBS. Nuclei were stained with H 33258. Stained sections were washed three times in PBS for 5 min and mounted in Fluormount G. Ventral horn motor neurons were counted in 6 ventral horns per animal in 20× epifluorescence images using ImageJ. For quantification of axonal densities, confocal images (63×) of corticospinal tract and dorsal column were taken and axonal densities were analysed in these regions by counting at least 500 axons per region with ImageJ.

### ^3^H-Thymidine incorporation assay

Single cell suspensions were prepared from a pool of mesenteric, axial and brachial lymph nodes of *Ngr1/2* ^−/−^ animals or WT controls eight days after immunization. Lymph node cells from immunized animals were cultured in 96 well plates at 2×10^5^ cells/ well in RPMI supplemented with 10% FCS and 50 µM 2-mercaptoethanol and stimulated with different concentrations of MOG 35–55 peptide or 0.1 µg/ ml anti-CD3 (145-2C11). After two days, cells were pulsed with 1 µCi [methyl-^3^H]-Thymidine (Amersham) per well for 16 h. Cells were harvested and spotted on filtermats using Harvester 96 MACH III M (Tomtec) according to manufacturer's instructions. Spotted filtermats were dried and sealed in bags containing betaplate scintillation liquid (Perkin-Elmer). Incorporated activity/ 96 well was assessed in a beta counter (1450 Microbeta, Perkin-Elmer) in counts per minute (cpm). Stimulation index of applied peptides or antibodies was calculated by dividing the mean incorporated activity of stimulated wells by the mean of unstimulated control wells.

### Flow cytometry

Single cell suspensions of splenocytes were prepared by passing of tissue through a 40 µm cell strainer (BD Falcon). For isolation of CNS-infiltrating leukocytes, mice were perfused transcardially with 10 ml PBS. Brain and spinal cord were removed, minced and digested with a solution of Collagenase/ DNaseI (Roche Applied Science) in D-MEM for 30 min at 37°C. The digested tissue was triturated by passage through a 40 µm cell strainer (BD Falcon). CNS-infiltrating cells were separated from myelin debris by percoll gradient centrifugation (30%/ 78%). Cells were recovered from the interface. For detection of intracellular cytokines, cells were stimulated with PMA/ Ionomycin (both Sigma-Alrich) for 5 h in the presence of Brefeldin A (eBioscience). Flow cytometric staining was performed in FACS-Buffer (0.5% BSA, 0.02% NaN_3_, PBS) in the presence of anti-CD16/32 (Fc-Block) (93). For quantification of CNS-infiltrating leukocytes, ten percent of the isolated cell suspension was analyzed using TruCount tubes (BDBiosciences) in combination with anti-CD45 staining. Fixation and permeabilization for intracellular staining of cytokines or FoxP3 were performed using IC fixation and permeabilization kit or FoxP3 staining buffer set (both eBioscience) according to manufacturer's instructions. For intracellular cytokine staining, dead cells were excluded from the analysis using aqua-live/dead fixable cell stain kit (Molecular Probes). Flow cytometric analysis was performed using the following antibodies: anti-CD3e-PacificBlue (500A2), anti-CD3e-PerCPCy5.5 (145-2C11), anti-CD4-FITC (GK1.5), anti-CD4-PacificBlue (GK1.5), anti-CD8a-PacificBlue (53-6.7), anti-CD8a-PECy7 (53-6.7), anti-CD11b-FITC (M1/70), anti-CD11b-PerCPCy5.5 (M1/70), anti-CD11c-APC (N418), anti-CD11c-PECy7 (N418), anti-CD25-APC (PC61.5), anti-CD45-APC-eFluor750 (30-F11), anti-CD45R(B220)-PECy5.5 (RA3-6B2), anti-CD69-FITC (H1.2F3), anti-CD80-PE (16-10A1), anti-CD138-APC (281-2), anti-FoxP3-PE (FJK-16s), anti-IFN-γ-PE (XMG1.2), anti-IL-4-PE (11B11), anti-IL-10-APC (JES5-16E3), anti-IL-17A (eBio17B7), anti-Ly-6G-PE (1A8), anti-Ly-6G-PacificBlue (1A8), anti-MHCII-FITC (M5/ 115.15.2) and anti-NK1.1-PECy7 (PK138).

Samples were analyzed on a LSRII flow cytometer (BD) using appropriate compensation controls and doublet discrimination.

### Brain Tissue Homogenization

C57BL/6 and *Ngr1/2* ^−/−^ mice were sacrificed during acute disease (13 days after immunization), brains were removed and shock frozen in liquid nitrogen. Brains were roughly cut in half and homogenized in modified RIPA buffer (150 mM NaCl, 50 mM TRIS-HCl, 1% sodium deoxycholate, 1% Triton X-100, 1 mM EDTA). Protease inhibitor cocktail (Roche, Mannheim, Germany) and phenylmethanesulfonylfluoride (PMSF, 0.1 M in isopropanol) were added to the lysis buffer immediately before tissue homogenization. 1 ml lysis buffer was added per each half brain and the tissue was homogenized on ice for 1 minute at high speed using a T8 Ultra-turrax mixer (IKA Werke GmbH & Co. KG, Germany). Samples were then centrifuged at 5000 rpm for 30 minutes at 4°C. Total protein concentration was determined in the resulting supernatant with the bicinchoninic acid assay. Samples were aliquoted and stored at −80°C.

### ELISA

Serum IgG antibodies to human recombinant MOG Ig (1–125) were analyzed by ELISA as described before [36]. Mouse sera were diluted 1∶100. Bound antibodies were detected by a HRP-coupled anti-mouse IgG antibody (GE Healthcare Bio-Sciences, Uppsala, Sweden).

### Flow Cytomix

IL-6, IL-12p70, TGF-β1, TNF-α, IL-10, IL-17, IFN-γ, IL-5, and IL-4 concentrations were quantified in brain lysates using a cytofluorimetry-based ELISA system (FlowCytomix, Bender Medsystems Gmbh, Austria). In brief, brains were homogenized in lysis buffer as described above, and stored at −80°C. Samples were thawed on ice and cytokine concentrations were determined according to the manufacturer's instructions.

### Statistical analysis

Two-tailed unpaired student's t-test was used to compare the number of CNS-infiltrating leukocytes between *Ngr1/2* ^−/−^ mice and WT controls. Two-way ANOVA was used to analyse EAE scores (repeated measures) and neuronal and axonal loss (not repeated measures). Two-way ANOVA (repeated measures) combined with Bonferroni post-analysis was used to analyse different immune cell subsets between experimental groups of *Ngr1/2* ^−/−^ mice and WT controls.

## References

[1] Compston A, Coles A (2008). Multiple sclerosis.. Lancet.

[2] Sospedra M, Martin R (2005). Immunology of multiple sclerosis.. Annu Rev Immunol.

[3] Kornek B, Storch MK, Weissert R, Wallstroem E, Stefferl A (2000). Multiple sclerosis and chronic autoimmune encephalomyelitis: a comparative quantitative study of axonal injury in active, inactive, and remyelinated lesions.. Am J Pathol.

[4] Lovas G, Szilagyi N, Majtenyi K, Palkovits M, Komoly S (2000). Axonal changes in chronic demyelinated cervical spinal cord plaques.. Brain.

[5] Tallantyre EC, Bo L, Al-Rawashdeh O, Owens T, Polman CH Clinico-pathological evidence that axonal loss underlies disability in progressive multiple sclerosis.. Mult Scler.

[6] Schwab ME (2010). Functions of Nogo proteins and their receptors in the nervous system.. Nat Rev Neurosci.

[7] Silver J, Miller JH (2004). Regeneration beyond the glial scar.. Nat Rev Neurosci.

[8] Moore DL, Blackmore MG, Hu Y, Kaestner KH, Bixby JL (2009). KLF family members regulate intrinsic axon regeneration ability.. Science.

[9] Fournier AE, GrandPre T, Strittmatter SM (2001). Identification of a receptor mediating Nogo-66 inhibition of axonal regeneration.. Nature.

[10] Liu BP, Fournier A, GrandPre T, Strittmatter SM (2002). Myelin-associated glycoprotein as a functional ligand for the Nogo-66 receptor.. Science.

[11] Wang KC, Koprivica V, Kim JA, Sivasankaran R, Guo Y (2002). Oligodendrocyte-myelin glycoprotein is a Nogo receptor ligand that inhibits neurite outgrowth.. Nature.

[12] Venkatesh K, Chivatakarn O, Lee H, Joshi PS, Kantor DB (2005). The Nogo-66 receptor homolog NgR2 is a sialic acid-dependent receptor selective for myelin-associated glycoprotein.. J Neurosci.

[13] Atwal JK, Pinkston-Gosse J, Syken J, Stawicki S, Wu Y (2008). PirB is a functional receptor for myelin inhibitors of axonal regeneration.. Science.

[14] Goh EL, Young JK, Kuwako K, Tessier-Lavigne M, He Z (2008). beta1-integrin mediates myelin-associated glycoprotein signaling in neuronal growth cones.. Mol Brain.

[15] Hu F, Strittmatter SM (2008). The N-terminal domain of Nogo-A inhibits cell adhesion and axonal outgrowth by an integrin-specific mechanism.. J Neurosci.

[16] Pool M, Niino M, Rambaldi I, Robson K, Bar-Or A (2009). Myelin regulates immune cell adhesion and motility.. Exp Neurol.

[17] Fry EJ, Ho C, David S (2007). A role for Nogo receptor in macrophage clearance from injured peripheral nerve.. Neuron.

[18] Satoh J, Onoue H, Arima K, Yamamura T (2005). Nogo-A and nogo receptor expression in demyelinating lesions of multiple sclerosis.. J Neuropathol Exp Neurol.

[19] David S, Fry EJ, Lopez-Vales R (2008). Novel roles for Nogo receptor in inflammation and disease.. Trends Neurosci.

[20] Fontoura P, Ho PP, DeVoss J, Zheng B, Lee BJ (2004). Immunity to the extracellular domain of Nogo-A modulates experimental autoimmune encephalomyelitis.. J Immunol.

[21] Karnezis T, Mandemakers W, McQualter JL, Zheng B, Ho PP (2004). The neurite outgrowth inhibitor Nogo A is involved in autoimmune-mediated demyelination.. Nat Neurosci.

[22] Yang Y, Liu Y, Wei P, Peng H, Winger R Silencing Nogo-A promotes functional recovery in demyelinating disease.. Ann Neurol.

[23] Zheng B, Atwal J, Ho C, Case L, He XL (2005). Genetic deletion of the Nogo receptor does not reduce neurite inhibition in vitro or promote corticospinal tract regeneration in vivo.. Proc Natl Acad Sci U S A.

[24] Worter V, Schweigreiter R, Kinzel B, Mueller M, Barske C (2009). Inhibitory activity of myelin-associated glycoprotein on sensory neurons is largely independent of NgR1 and NgR2 and resides within Ig-Like domains 4 and 5.. PLoS One.

[25] McDonald CL, Steinbach K, Kern F, Schweigreiter R, Martin R Nogo receptor is involved in the adhesion of dendritic cells to myelin.. J Neuroinflammation.

[26] Zhang L, Zheng S, Wu H, Wu Y, Liu S (2009). Identification of BLyS (B lymphocyte stimulator), a non-myelin-associated protein, as a functional ligand for Nogo-66 receptor.. J Neurosci.

[27] Moore PA, Belvedere O, Orr A, Pieri K, LaFleur DW (1999). BLyS: member of the tumor necrosis factor family and B lymphocyte stimulator.. Science.

[28] Gross JA, Johnston J, Mudri S, Enselman R, Dillon SR (2000). TACI and BCMA are receptors for a TNF homologue implicated in B-cell autoimmune disease.. Nature.

[29] Gross JA, Dillon SR, Mudri S, Johnston J, Littau A (2001). TACI-Ig neutralizes molecules critical for B cell development and autoimmune disease. impaired B cell maturation in mice lacking BLyS.. Immunity.

[30] Di Lorenzo A, Manes TD, Davalos A, Wright PL, Sessa WC (2011). Endothelial Reticulon-4B (Nogo-B) regulates ICAM-1-mediated leukocyte transmigration and acute inflammation.. Blood.

[31] Yu J, Fernandez-Hernando C, Suarez Y, Schleicher M, Hao Z (2009). Reticulon 4B (Nogo-B) is necessary for macrophage infiltration and tissue repair.. Proc Natl Acad Sci U S A.

[32] Wright PL, Yu J, Di YP, Homer RJ, Chupp G (2010). Epithelial reticulon 4B (Nogo-B) is an endogenous regulator of Th2-driven lung inflammation.. J Exp Med.

[33] Schanda K, Hermann M, Stefanova N, Gredler V, Bandtlow C (2011). Nogo-B is associated with cytoskeletal structures in human monocyte-derived macrophages.. BMC Res Notes.

[34] Miao RQ, Gao Y, Harrison KD, Prendergast J, Acevedo LM (2006). Identification of a receptor necessary for Nogo-B stimulated chemotaxis and morphogenesis of endothelial cells.. Proc Natl Acad Sci U S A.

[35] Voskuhl RR, Peterson RS, Song B, Ao Y, Morales LB (2009). Reactive astrocytes form scar-like perivascular barriers to leukocytes during adaptive immune inflammation of the CNS.. J Neurosci.

[36] Wang H, Munger KL, Reindl M, O'Reilly EJ, Levin LI (2008). Myelin oligodendrocyte glycoprotein antibodies and multiple sclerosis in healthy young adults.. Neurology.

